# Genomic characterization of *Streptococcus equi* subspecies *zooepidemicus* from a 2021 outbreak in Indiana with increased sow mortality

**DOI:** 10.1128/msphere.00404-23

**Published:** 2023-10-20

**Authors:** Xuhua Chen, Kathy Mou, Weili Lu, Loni Schumacher, Nubia Resende-De-Macedo, Panchan Sitthicharoenchai, Rachel Derscheid, Eric Burrough, Ganwu Li

**Affiliations:** 1 Department of Veterinary Diagnostic and Production Animal Medicine, College of Veterinary Medicine, Iowa State University, Ames, Iowa, USA; 2 Department of Population Health and Pathobiology, College of Veterinary Medicine, North Carolina State University, Raleigh, North Carolina, USA; University of Napoli Federico II, Naples, Italy

**Keywords:** *Streptococcus equi *subspecies *zooepidemicus*, *S. zooepidemicus*, whole-genome sequencing

## Abstract

*Streptococcus equi* subspecies *zooepidemicus* (*S. zooepidemicus*) was reported to cause epizootic outbreaks in swine, resulting in significant economic losses in Asia in the 1970s and in North America in 2019. In February 2021, another outbreak due to *S. zooepidemicus* occurred in 2-year-old adult sows in a production system in Indiana. Whole-genome sequencing analysis of three outbreak isolates from Indiana revealed that all were genetically distant from the isolates of a strain that caused high mortality events in Ohio and Tennessee in 2019, as well as from the ATCC 35246 strain that caused outbreaks in China in the 1970s. The Indiana outbreak isolates were also distantly related to outbreak-unrelated swine isolate AZ-45470 from Arizona but closely related to an *S. zooepidemicus* equine isolate from Iowa. Comparative genomic analysis revealed that one genomic island, GI-13, was unique to the genome of a representative Indiana outbreak isolate, IN-6992. The genomic islands GI-1, GI-3, and GI-10 were also present in IN-6992 but absent from the Ohio and Tennessee outbreak isolates. In addition, the ATCC 35246-type *szM* gene and the Fic domain-containing protein gene (*bifA*) were absent from IN-6992, while present in all isolates from the Ohio and Tennessee outbreaks. Our findings have significant implications for understanding, monitoring, and developing control measures against diseases caused by *S. zooepidemicus*.

**IMPORTANCE:**

This study highlights a *Streptococcus equi* subspecies *zooepidemicus* (*S. zooepidemicus*) strain isolated from an outbreak in Indiana, which resulted in mortality events among a swine herd in 2021. The Indiana outbreak strain was found to be genetically and phylogenetically distant to a strain isolated from the 2019 outbreaks in Ohio and Tennessee, which caused high swine mortality. We also discovered multiple unique genetic features in the Indiana outbreak strain, including distinct *S. zooepidemicus* genomic islands, and notable *S. zooepidemicus* virulence genes—many of which could serve as biomarkers for the diagnosis of this strain. These findings provide significant insights into monitoring and potentially preventing severe outbreaks caused by the Indiana outbreak strain in the future.

## OBSERVATION

*Streptococcus equi* subspecies *zooepidemicus* (*S. zooepidemicus*) is a beta-hemolytic, Gram-positive bacterium frequently isolated as an opportunistic pathogen from horses, while also causing infections in many other animal species (1). In the 1970s, *S. zooepidemicus* reportedly caused epizootic outbreaks in swine populations in China and Indonesia, resulting in significant economic losses (2, 3); however, isolation of *S. zooepidemicus* from clinically ill pigs in the US has been historically limited. Canada was the first to report a high mortality event in North American swine due to *S. zooepidemicus* in March 2019 (4). A half year later, three outbreak cases of *S. zooepidemicus* occurred in culled sows and feeder pigs in Ohio, Tennessee, and Pennsylvania, with mortalities of up to 50% (1, 5). Whole-genome sequencing analysis revealed that the outbreak isolates from Ohio and Tennessee clustered together with the prototype strain ATCC 35246, which caused high mortality outbreaks in China in the 1970s (6). However, the Ohio and Tennessee outbreak isolates differed significantly from an outbreak-unrelated swine isolate from Arizona and isolates from horses and other animal species.

**Fig 1:**
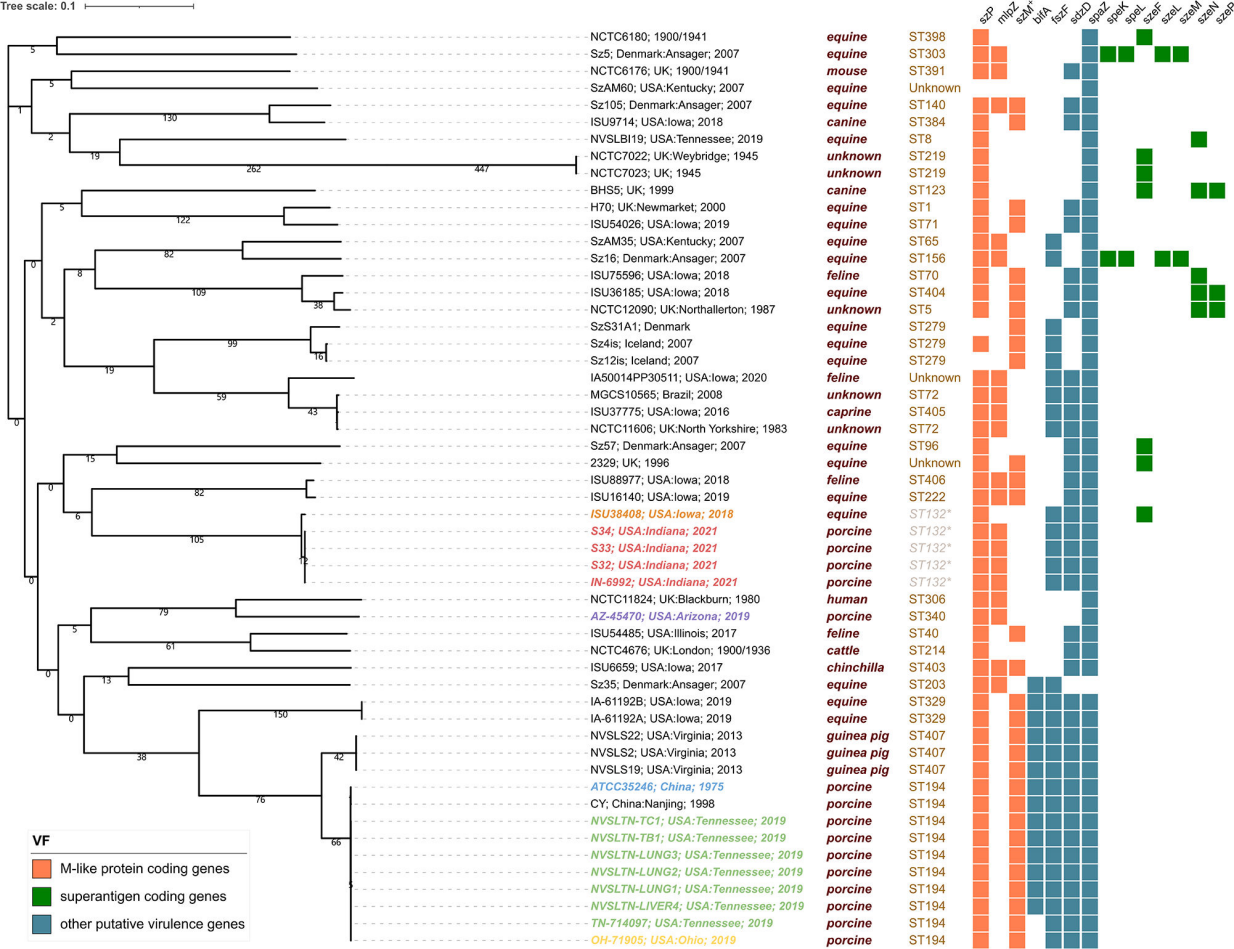
Whole-genome sequence-based phylogenetic analysis was conducted using SNPs located in 55 tested *S. zooepidemicus* genomes to generate a core SNP parsimony tree. The branches of the tree are proportional to the distance between the isolates. The branch labels contain the year and geographical origin of each isolate. The right side of the figure lists the host information, MLST, and the distribution of putative virulence genes classified into three groups: (i) M-like protein-coding genes (orange), (ii) other putative virulence genes (blue), and (iii) superantigen-coding genes (green). * indicates that the isolates lacked the key housekeeping gene *yqiL*, and thus their MLST types were determined by SNPs from the remaining six housekeeping genes. + indicates that the *szM* in the figure represents ATCC 35246-type *szM*.

In spring 2021, 2-year-old adult sows from a 2,400 sow herd in Indiana experienced an increased death loss, resulting in 66 deaths within a 6-week period (2.75% mortality). Reported clinical signs included cyanotic ears, abortion, and uterine discharge. Submission of fresh and fixed tissues (heart, lung, liver, uterus, and kidney) to the Iowa State University Veterinary Diagnostic Laboratory on 14 January 2021 revealed lesions of septicemia in the lungs and kidney. RT-PCR/PCR techniques were employed to exclude the presence of the porcine reproductive and respiratory syndrome virus, the swine influenza virus, and the bacterial pathogen *Glaesserella parasuis. S. zooepidemicus* was isolated, and whole-genome sequencing of three isolates was performed on the Illumina MiSeq platform as previously published (1). More than 90% of the processed paired-end reads had a Phred score of 35, which indicated high-quality sequence data. The *de novo* assembly of raw reads generated contigs with total sizes ranging from 2.0 to 2.2 Mbp. The overall GC content was between 41% and 42%, which was similar to the reference genome ATCC 35246 (2,167,164 bp, 41.65% GC) (6) and Ohio and Tennessee outbreak isolates (41.65%) (1). Interestingly, all Indiana outbreak isolates lacking a key housekeeping gene (for determining their multilocus sequence typing [MLST] types), *yqiL,* were closely related to MLST type ST132; in contrast, the Ohio and Tennessee outbreak isolates and the ATCC 35246 strain all fell under ST194. Whole-genome phylogenetic analysis based on core genome single-nucleotide polymorphisms (SNPs) was conducted using previous methods (1). The analysis consisted of 55 *S*. *zooepidemicus* isolates, including 31 sequenced isolates and 24 strains from different countries with publicly available genome sequences (Table S1) (1). Using kSNP3, a total of 117,897 SNPs were identified from all the isolates. The phylogenetic analysis based on whole genome SNPs showed that outbreak isolates from Indiana were genetically distant from the Ohio and Tennessee outbreak isolates as well as ATCC 35246 (Fig. 1). The three Indiana outbreak isolates were also phylogenetically distant from the outbreak-unrelated swine isolate from Arizona, AZ-45470, but were most closely related to ISU38408, an *S. zooepidemicus* isolate from a horse in Iowa in 2018.

One of the three Indiana outbreak isolates, IN-6992, was selected along with its most closely related isolate, ISU38408, for long-read sequencing using Oxford Nanopore Technologies (1). Full-length genome sequences were successfully obtained from a hybrid assembly of Illumina short reads and Nanopore long reads (1). Both *S. zooepidemicus* strains IN-6992 and ISU38408 had identical average GC content of 41.6% and contained circular chromosomes of 2,156,971 and 2,079,123 bp, respectively (Table S2). Comparative genomic analysis was performed and visualized with BLAST Ring Image Generator as previously described (1) to generate a circular genomic map of IN-6992 and ISU38408 closed genome sequences, and five additional complete sequences of control strains: ATCC 35246 and CY strains from China (GenBank accession numbers: CP002904.1 and CP006770.1), and OH-71905, TN-714097, and AZ-45470 from the USA (GenBank accession numbers: CP046040.1, CP046042.2, and CP046041.1) (Fig. S1). Comparative analysis of IN-6992 with OH-71905, TN-74097, and ATCC 35246 showed that IN-6992 had an average nucleotide identity of 97.2% with the three control strains, and 88.8% of nucleotides of IN-6992 aligned with the three strains. Compared to AZ-45470, IN-6992 had an average nucleotide identity of 97.3%, and 87.2% of its nucleotides aligned with AZ-45470. In addition, the alignment performed in IN-6992 and ISU38408 showed an average nucleotide identity of 99.9%, and 96.1% of nucleotides of IN-6992 aligned with ISU38408. When the genomes of IN-6992 and ISU38408 were compared, 1,135 SNPs, 17 insertions, and 8 deletions were found in the genome of IN-6992.

**Fig 2:**
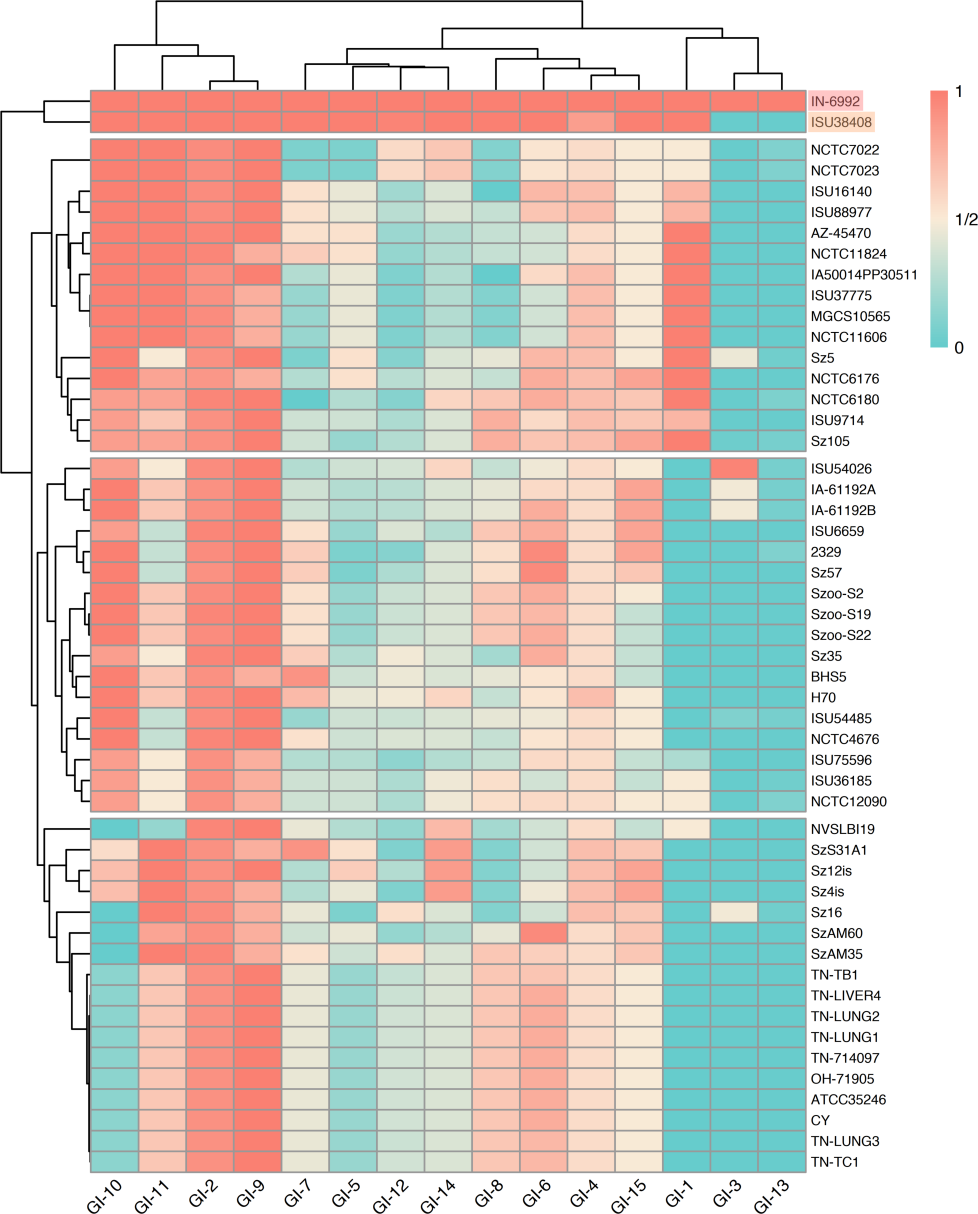
Two-way clustering of GI prevalence among *S. zooepidemicus* isolates. A red–white–blue heat map was constructed based on the percentage of the coding sequences (CDS) (0 = 0%, 1 = 100%) in each predicted IN-6992 GI that was also present in all *S. zooepidemicus* isolates. Clustering was performed to illustrate the similarities between the prevalence of the GIs examined and between the *S. zooepidemicus* isolates regarding CDS proportion. The red-highlighted isolate at the top right indicates IN-6992, while the orange-highlighted isolate (top right) indicates the horse isolate ISU38408 that was closely related to IN-6992.

Genomic islands (GIs) were also predicted in IN-6992 following previous methods (1). Fifteen GIs with significantly different GC content compared to the core genome of IN-6992 were identified, with sizes varying from 4 to 56 kb (Fig. 2; Table S3). The genomic island GI-13 was found only in IN-6992 out of all isolates included in this study. Therefore, the genes encoded by this genomic island could serve as biomarkers for this strain of *S. zooepidemicus*. In addition, GI-1, GI-3, and GI-10 were present in IN-6992 but not in the Ohio and Tennessee outbreak isolates. However, GI-2 and GI-9 were present in nearly all *S. zooepidemicus* isolates (> 91.6% and >77.8%, respectively). Several GIs encoding putative virulence genes could possibly contribute to the virulence and host adaptation of *S. zooepidemicus* (Table S4). GI-1 encoded a putative ABC transporter system and putative regulator; GI-10 encoded a bacteriocin and its secretion system; GI-11 encoded a glycohydrolase toxin TNT-related protein; GI-12 encoded a zeta toxin family protein and a type IV secretion system protein; and GI-15 encoded a putative holin-like toxin. The pathogenic role of these GIs requires further study.

The presence of 15 previously reported putative virulence genes (1) was examined and compared among all 55 *S*. *zooepidemicus* isolates in this study (Fig. 1; Table S5). All isolates had a *szM* gene, and the nucleotide identities within the *szM* gene sequences ranged from 44.1% to 99.9%. Specifically, the *szM* gene sequence exhibited 48.3% nucleotide identity with OH71905, TN714097, and ATCC35246 strains, and 47.2% nucleotide identity with AZ-45470, while showing 100% nucleotide with ISU38408. For a visual representation, a phylogenetic tree of all *szM* genes is displayed in Figure S2. The gene *bifA* was absent from the Indiana outbreak isolate, but present in all eight isolates from Ohio and Tennessee outbreaks. In contrast, the M-like protein gene *mlpZ* was absent in all tested Ohio and Tennessee outbreak isolates but was present in all three Indiana outbreak isolates. Several superantigen genes, including *szeF*, *szeL*, *szeM*, *szeN,* and *szeP* (7–10), were not detected in the Indiana outbreak isolate, Ohio and Tennessee outbreak isolates (1), or the ATCC 35246 and CY strains from outbreaks in China, suggesting that they are not necessary for the virulence.

In summary, genomic epidemiological analysis of *S. zooepidemicus* isolates from a recent outbreak in Indiana revealed significant genetic distinctions compared to previous outbreak isolates that caused high mortality events in Ohio and Tennessee in 2019 and a strain that caused outbreaks in China in the 1970s. Comparative genomic analysis also identified unique genomic islands and the presence/absence of specific virulence genes that further distinguished this Indiana strain from others. Our findings provide important insight for future investigations of the virulence mechanisms and evolution of *S. zooepidemicus*, guiding the development of better tracking and control measures for the prevention of severe *S. zooepidemicus* outbreaks.

## Data Availability

The complete genome sequences of isolates IN-6992 and ISU38408 are available at NCBI under BioProject accession PRJNA588803 (accession numbers CP073275.1 and CP074115.1).

## SUPPLEMENTAL MATERIAL

Supplemental material msphere.00404-23-s0001.pdf Supplemental figures and tables.

ASM does not own the copyrights to Supplemental Material that may be linked to, or accessed through, an article. The authors have granted ASM a non-exclusive, world-wide license to publish the Supplemental Material files. Please contact the corresponding author directly for reuse.
