# Supplementary material for: Genomic characterization of Streptococcus equi subspecies zooepidemicus from a 2021 outbreak in Indiana with increased sow mortality
Source: mSphere. 2023 Oct 20;8(6):e00404-23. doi: 10.1128/msphere.00404-23 (PMC10732033; doi:10.1128/msphere.00404-23)
Supplement: Supplemental material — Supplemental figures and tables. [file msphere.00404-23-s0001.pdf]

Supplemental Material

Figure S1. Comparative genome analysis among 7 *S. zooepidemicus* isolates

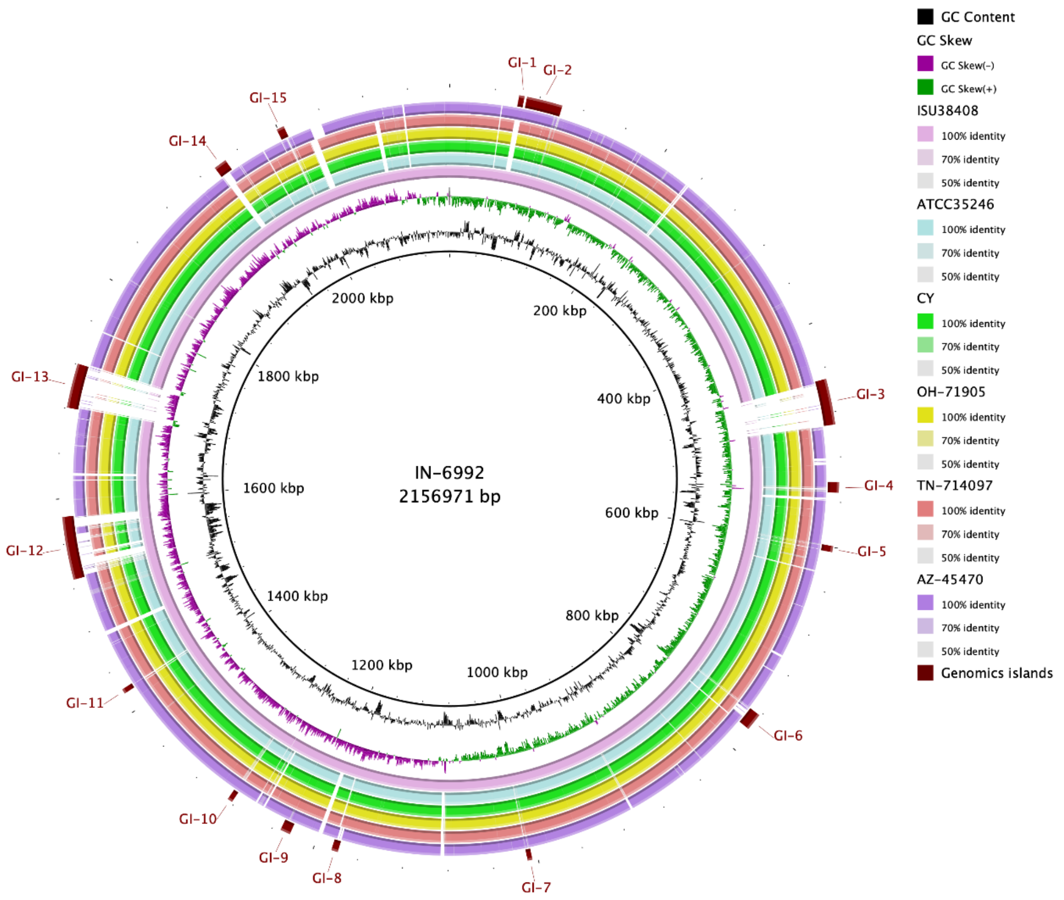

Figure S2. Phylogenetic analysis of *szM* gene sequences from Fifty-five *S. zooepidemicus* isolates included in the study

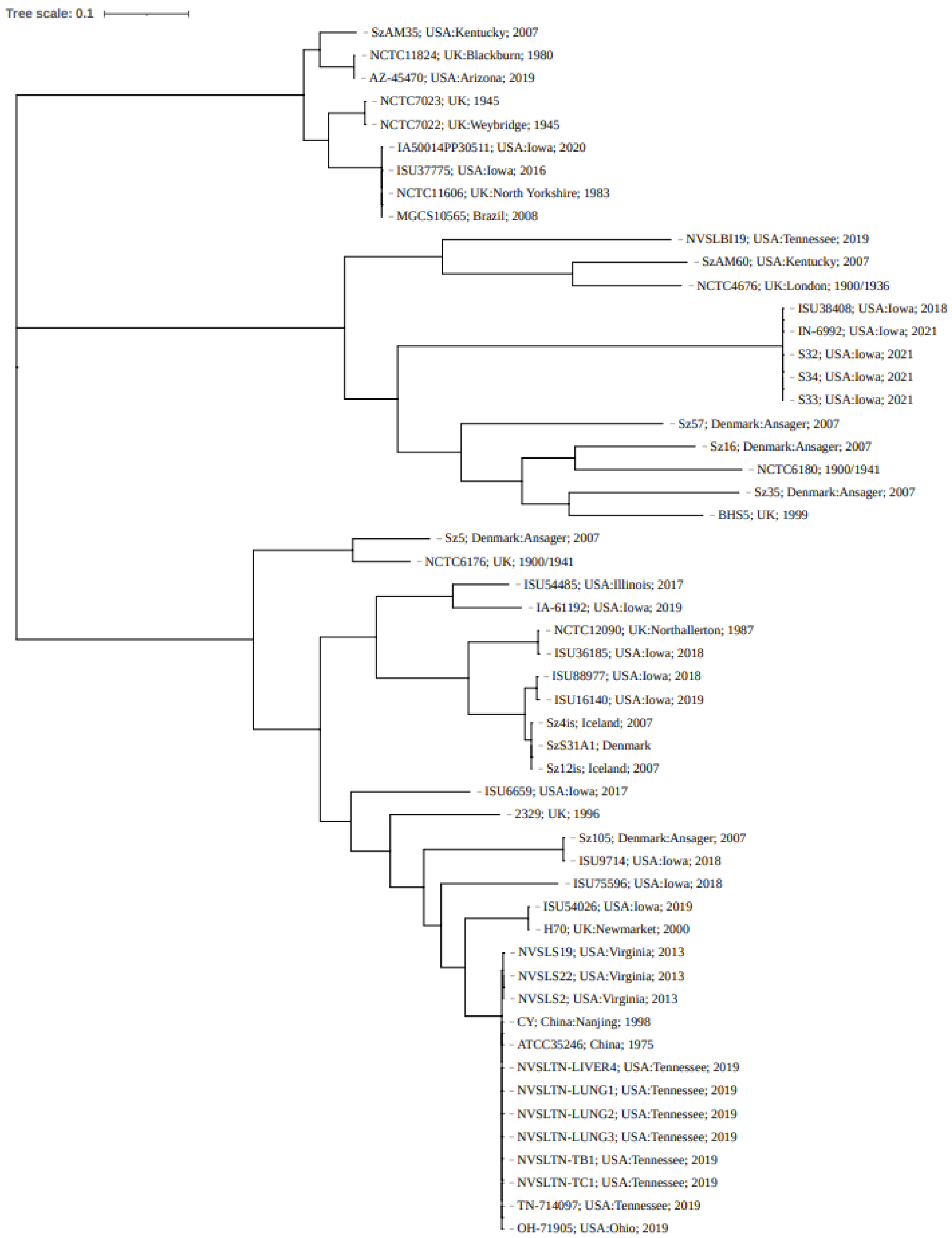

Table S1. Fifty-five *S. zooepidemicus* isolates included in the study

isolates	host	date	country	accession	assembly level
2329	equine	1996	United Kingdom	GCA_000836615.1	Contig
ATCC 35246	porcine	1975	China	CP002904.1	Complete Genome
BHS5	canine	1999	United Kingdom	GCA_000208725.1	Scaffold
CY	porcine	1998	China: Nanjing	CP006770.1	Complete Genome
H70	equine	2000	United Kingdom:Newmarket	FM204884.1	Complete Genome
MGCS10565		2008	Brazil	CP001129.1	Complete Genome
NCTC4676	cattle	1900/1936	United Kingdom: London	GCA_900459475.1	Contig
NCTC6176	mouse	1900/1941	United Kingdom	LS483368.1	Complete Genome
NCTC6180	equine	1900/1941		LR134317.1	Complete Genome
NCTC7022		1945	United Kingdom: Weybridge	LS483325.1	Complete Genome
NCTC7023		1945	United Kingdom	GCA_900460185.1	Contig
NCTC11606		1983	United Kingdom: North Yorkshire	LS483354.1	Complete Genome
NCTC11824	human	1980	United Kingdom: Blackburn	LS483380.1	Complete Genome
NCTC12090		1987	United Kingdom: Northallerton	LS483328.1	Complete Genome
Sz4is	equine	2007	Iceland	GCA_000876215.1	Contig
Sz5	equine	2007	Denmark:Ansager	GCA_000876355.1	Contig
Sz12is	equine	2007	Iceland	GCA_000876305.1	Contig
Sz16	equine	2007	Denmark:Ansager	GCA_000876295.1	Contig
Sz35	equine	2007	Denmark:Ansager	GCA_000876365.1	Contig
Sz57	equine	2007	Denmark:Ansager	GCA_000876375.1	Contig
Sz105	equine	2007	Denmark:Ansager	GCA_000876195.1	Contig
SzAM35	equine	2007	USA: Lexington, Kentucky	GCA_000876275.1	Contig

SzAM60	equine	2007	USA: Lexington, Kentucky	GCA_000876285.1	Contig
SzS31A1	equine		Denmark	GCA_000445225.2	Contig
ISU37775	caprine	2016	USA: Iowa	GCA_011316805.1	Contig
ISU6659	chinchilla	2017	USA: Iowa	GCA_011316715.1	Contig
ISU54485	feline	2017	USA: Illinois	GCA_011316825.1	Contig
ISU9714	canine	2018	USA: Iowa	GCA_010994195.1	Contig
ISU36185	equine	2018	USA: Iowa	GCA_011316755.1	Contig
ISU38408	equine	2018	USA: Iowa	CP074115.1	Complete Genome
ISU75596	feline	2018	USA: Iowa	GCA_011316835.1	Contig
ISU88977	feline	2018	USA: Iowa	GCA_011316885.1	Contig
ISU16140	equine	2019	USA: Iowa	GCA_011316745.1	Contig
ISU54026	equine	2019	USA: Iowa	GCA_011316855.1	Contig
AZ-45470	porcine	2019	USA: Arizona	CP046041.1	Complete Genome
IA-61192	equine	2019	USA: Iowa	GCA_011317105.1	Contig
OH-71905	porcine	2019	USA: Ohio	CP046040.1	Complete Genome
TN-74097	porcine	2019	USA: Tennessee	CP046042.1	Complete Genome
NVSLBI19	equine	2019	USA: Tennessee	GCA_011316705.1	Contig
NVSLTN-LIVER4	porcine	2019	USA: Tennessee	GCA_011317085.1	Contig
NVSLTN-LUNG1	porcine	2019	USA: Tennessee	GCA_011317015.1	Contig
NVSLTN-LUNG2	porcine	2019	USA: Tennessee	GCA_011317045.1	Contig
NVSLTN-LUNG3	porcine	2019	USA: Tennessee	GCA_011317035.1	Contig
NVSLTN-TB1	porcine	2019	USA: Tennessee	GCA_011317005.1	Contig
NVSLTN-TC1	porcine	2019	USA: Tennessee	GCA_011316985.1	Contig
NVSLVA-S2	guinea pig	2013	USA: Virginia	GCA_011316925.1	Contig
NVSLVA-S19	guinea pig	2013	USA: Virginia	GCA_011316915.1	Contig

NVSLVA-S22	guinea pig	2013	USA: Virginia	GCA_011316905.1	Contig
IA50014PP30511	feline	2020	USA: Iowa		Contig
IN-6992	porcine	2021	USA: Indiana	CP073275.1	Complete Genome
S32	porcine	2021	USA: Indiana		Contig
S33	porcine	2021	USA: Indiana		Contig
S34	porcine	2021	USA: Indiana		Contig

Table S2. Genomic features of *S. zooepidemicus* isolates IN-6992 (Representative Indiana outbreak isolate) and ISU38408

Feture	IN-6992	ISU38408
Length, bp	2,156,971	2,079,123
GC Content	41.57%	41.65%
Genes (total)	2,073	1,971
CDSs (total)	1,984	1,882
rRNAs	6, 6, 6 (5S, 16S, 23S)	6, 6, 6 (5S, 16S, 23S)
tRNAs	67	67
ncRNAs	4	4
Pseudo Genes (total)	58	62

*CDS, coding sequence; tRNA, transfer RNA; rRNA, ribosomal RNA; ncRNA, non-coding RNA.

Table S3. Total CDS present or absent within each predicted genomic island of *S. zooepidemicus* strains.

An identity and coverage cutoff of 75% were used to determine the presence or absence of genomic islands. (CDSs > 75%, with more than 75% of the CDS in a GI present)

	GI-1	GI-2	GI-3	GI-4	GI-5	GI-6	GI-7	GI-8	GI-9	GI-10	GI-11	GI-12	GI-13	GI-14	GI-15
**Total CDS**	4	36	50	7	11	19	11	9	9	7	6	52	58	15	6
2329	0/4	34/36	1/50	4/7	1/11	18/19	7/11	5/9	9/9	7/7	2/6	6/52	6/58	6/15	5/6
ATCC35246	0/4	33/36	1/50	4/7	2/11	15/19	5/11	6/9	9/9	1/7	4/6	19/52	1/58	6/15	3/6
AZ-45470	4/4	35/36	1/50	4/7	6/11	7/19	6/11	3/9	9/9	7/7	6/6	10/52	1/58	4/15	3/6
BHS5	0/4	33/36	0/50	4/7	4/11	10/19	10/11	4/9	7/9	7/7	4/6	24/52	1/58	6/15	2/6
CY	0/4	33/36	1/50	4/7	2/11	15/19	5/11	6/9	9/9	1/7	4/6	19/52	1/58	6/15	3/6
H70	0/4	34/36	1/50	5/7	5/11	10/19	8/11	3/9	9/9	7/7	4/6	25/52	1/58	9/15	3/6
IA-61192A	0/4	33/36	24/50	4/7	3/11	11/19	4/11	4/9	9/9	7/7	4/6	15/52	4/58	6/15	5/6
IA-61192B	0/4	33/36	24/50	4/7	3/11	15/19	4/11	4/9	9/9	7/7	4/6	15/52	4/58	6/15	5/6
IA50014PP30511	4/4	33/36	1/50	5/7	5/11	11/19	3/11	0/9	9/9	7/7	6/6	5/52	1/58	4/15	3/6
ISU16140	3/4	34/36	1/50	5/7	5/11	14/19	6/11	0/9	9/9	7/7	6/6	11/52	1/58	6/15	3/6
ISU36185	2/4	33/36	0/50	4/7	4/11	7/19	4/11	5/9	7/9	6/7	3/6	13/52	3/58	7/15	2/6
ISU37775	4/4	33/36	1/50	5/7	5/11	7/19	2/11	1/9	7/9	7/7	6/6	5/52	1/58	4/15	3/6
ISU38408	4/4	36/36	1/50	6/7	11/11	19/19	11/11	9/9	9/9	7/7	6/6	51/52	1/58	15/15	6/6
ISU54026	0/4	34/36	49/50	4/7	4/11	9/19	3/11	3/9	9/9	6/7	3/6	20/52	4/58	9/15	3/6
ISU54485	0/4	34/36	5/50	4/7	4/11	9/19	2/11	4/9	9/9	7/7	2/6	18/52	4/58	6/15	3/6
ISU6659	0/4	35/36	1/50	4/7	2/11	15/19	6/11	6/9	9/9	6/7	2/6	21/52	1/58	4/15	5/6
ISU75596	1/4	33/36	0/50	4/7	3/11	11/19	3/11	3/9	7/9	6/7	3/6	8/52	1/58	4/15	2/6
ISU88977	3/4	34/36	1/50	5/7	5/11	13/19	6/11	3/9	9/9	7/7	6/6	15/52	1/58	6/15	3/6
ISU9714	3/4	33/36	1/50	5/7	4/11	11/19	4/11	7/9	9/9	6/7	4/6	13/52	1/58	6/15	4/6
MGCS10565	4/4	34/36	1/50	5/7	5/11	7/19	2/11	1/9	7/9	7/7	6/6	5/52	1/58	4/15	3/6
NCTC11606	4/4	34/36	1/50	5/7	5/11	7/19	2/11	1/9	7/9	7/7	6/6	5/52	1/58	4/15	3/6
NCTC11824	4/4	35/36	1/50	4/7	6/11	7/19	7/11	3/9	7/9	7/7	6/6	9/52	1/58	4/15	3/6
NCTC12090	2/4	33/36	1/50	4/7	4/11	11/19	4/11	5/9	7/9	6/7	3/6	13/52	6/58	7/15	3/6

NCTC4676	0/4	35/36	1/50	4/7	4/11	10/19	6/11	3/9	9/9	7/7	2/6	21/52	1/58	6/15	3/6
NCTC6176	4/4	32/36	1/50	5/7	6/11	15/19	3/11	3/9	7/9	7/7	5/6	15/52	1/58	6/15	5/6
NCTC6180	4/4	35/36	1/50	5/7	3/11	15/19	0/11	6/9	9/9	6/7	5/6	6/52	5/58	9/15	4/6
NCTC7022	2/4	34/36	1/50	4/7	1/11	10/19	1/11	1/9	9/9	7/7	6/6	30/52	6/58	10/15	3/6
NCTC7023	2/4	34/36	1/50	4/7	1/11	10/19	1/11	1/9	9/9	7/7	6/6	30/52	6/58	10/15	3/6
NVSLBI19	2/4	35/36	0/50	4/7	3/11	7/19	5/11	2/9	9/9	0/7	1/6	9/52	1/58	11/15	2/6
OH-71905	0/4	33/36	1/50	4/7	2/11	15/19	5/11	6/9	9/9	1/7	4/6	19/52	1/58	6/15	3/6
Sz105	4/4	33/36	2/50	5/7	2/11	13/19	4/11	7/9	9/9	6/7	5/6	14/52	4/58	6/15	5/6
Sz12is	0/4	33/36	1/50	5/7	7/11	7/19	3/11	1/9	9/9	5/7	6/6	14/52	1/58	13/15	5/6
Sz16	0/4	34/36	24/50	5/7	1/11	7/19	5/11	1/9	7/9	0/7	6/6	29/52	3/58	6/15	4/6
Sz35	0/4	35/36	0/50	4/7	3/11	15/19	7/11	2/9	9/9	6/7	3/6	25/52	1/58	6/15	2/6
Sz4is	0/4	33/36	1/50	5/7	5/11	9/19	3/11	1/9	7/9	5/7	6/6	5/52	1/58	13/15	5/6
Sz57	0/4	33/36	0/50	4/7	1/11	18/19	7/11	5/9	9/9	7/7	2/6	13/52	1/58	6/15	4/6
Sz5	4/4	33/36	23/50	5/7	6/11	14/19	1/11	4/9	9/9	7/7	3/6	6/52	3/58	6/15	3/6
SzAM35	0/4	35/36	0/50	4/7	4/11	12/19	6/11	6/9	7/9	0/7	6/6	29/52	1/58	6/15	4/6
SzAM60	0/4	33/36	0/50	4/7	5/11	18/19	4/11	4/9	7/9	0/7	5/6	9/52	1/58	4/15	4/6
SzS31A1	0/4	33/36	0/50	5/7	6/11	7/19	10/11	1/9	7/9	4/7	6/6	5/52	1/58	13/15	4/6
Szoo-S19	0/4	35/36	1/50	4/7	2/11	14/19	6/11	6/9	9/9	7/7	4/6	18/52	1/58	6/15	2/6
Szoo-S22	0/4	34/36	1/50	4/7	2/11	15/19	6/11	6/9	9/9	7/7	4/6	18/52	1/58	6/15	2/6
Szoo-S2	0/4	34/36	1/50	4/7	2/11	15/19	6/11	6/9	9/9	7/7	4/6	18/52	1/58	6/15	3/6
TN-714097	0/4	33/36	1/50	4/7	2/11	15/19	5/11	6/9	9/9	1/7	4/6	19/52	1/58	6/15	3/6
TN-LIVER4	0/4	33/36	1/50	4/7	2/11	15/19	5/11	6/9	9/9	1/7	4/6	18/52	1/58	6/15	3/6
TN-LUNG1	0/4	33/36	1/50	4/7	2/11	15/19	5/11	6/9	9/9	1/7	4/6	19/52	1/58	6/15	3/6
TN-LUNG2	0/4	33/36	1/50	4/7	2/11	15/19	5/11	6/9	9/9	1/7	4/6	19/52	1/58	6/15	3/6
TN-LUNG3	0/4	33/36	1/50	4/7	2/11	14/19	5/11	6/9	9/9	1/7	4/6	18/52	1/58	6/15	3/6
TN-TB1	0/4	33/36	1/50	4/7	2/11	13/19	5/11	6/9	9/9	1/7	4/6	17/52	1/58	6/15	3/6
TN-TC1	0/4	33/36	1/50	4/7	2/11	14/19	5/11	6/9	9/9	1/7	4/6	19/52	1/58	6/15	3/6

Table S4. Predicted genomic islands and associated genes detected in IN-6992.

Locus	Gene ID	Length	Gene start	Gene end	Strand	Product
**GI-1**						
KCL43_00335		4889	61628	63220	1	ABC transporter ATP-binding protein
KCL43_00340		4889	63384	64502	1	ThiF family adenylyltransferase
KCL43_00345		4889	64495	65670	1	insulinase family protein
KCL43_00350		4889	65654	66517	-1	helix-turn-helix domain-containing protein
**GI-2**						
KCL43_00360		32160	68677	69978	1	MATE family efflux transporter
KCL43_00365	rpsJ	32160	70358	70666	1	30S ribosomal protein S10
KCL43_00370	rplC	32160	70964	71590	1	50S ribosomal protein L3
KCL43_00375	rplD	32160	71615	72238	1	50S ribosomal protein L4
KCL43_00380		32160	72238	72534	1	50S ribosomal protein L23
KCL43_00385	rplB	32160	72552	73385	1	50S ribosomal protein L2
KCL43_00390	rpsS	32160	73497	73775	1	30S ribosomal protein S19
KCL43_00395	rplV	32160	73791	74135	1	50S ribosomal protein L22
KCL43_00400	rpsC	32160	74148	74801	1	30S ribosomal protein S3
KCL43_00405	rplP	32160	74805	75218	1	50S ribosomal protein L16
KCL43_00410		32160	75228	75434	1	50S ribosomal protein L29
KCL43_00415	rpsQ	32160	75459	75722	1	30S ribosomal protein S17
KCL43_00420	rplN	32160	75747	76115	1	50S ribosomal protein L14
KCL43_00425	rplX	32160	76199	76504	1	50S ribosomal protein L24
KCL43_00430	rplE	32160	76528	77070	1	50S ribosomal protein L5
KCL43_00435		32160	77086	77271	1	type Z 30S ribosomal protein S14
KCL43_00440	rpsH	32160	77602	78000	1	30S ribosomal protein S8
KCL43_00445	rplF	32160	78323	78859	1	50S ribosomal protein L6

KCL43_00450	rplR	32160	78963	79319	1	50S ribosomal protein L18
KCL43_00455	rpsE	32160	79338	79832	1	30S ribosomal protein S5
KCL43_00460	rpmD	32160	79847	80029	1	50S ribosomal protein L30
KCL43_00465	rplO	32160	80230	80670	1	50S ribosomal protein L15
KCL43_00470	secY	32160	80687	81991	1	preprotein translocase subunit SecY
KCL43_00475		32160	82143	82784	1	adenylate kinase
KCL43_00480	infA	32160	82902	83120	1	translation initiation factor IF-1
KCL43_00485	rpmJ	32160	83146	83262	1	50S ribosomal protein L36
KCL43_00490	rpsM	32160	83280	83645	1	30S ribosomal protein S13
KCL43_00495	rpsK	32160	83663	84046	1	30S ribosomal protein S11
KCL43_00500		32160	84093	85031	1	DNA-directed RNA polymerase subunit alpha
KCL43_00505	rplQ	32160	85046	85432	1	50S ribosomal protein L17
KCL43_00510		32160	86737	88302	1	hypothetical protein
KCL43_00515		32160	88292	89167	1	hypothetical protein
KCL43_00520		32160	89188	89481	1	DUF4176 domain-containing protein
KCL43_00605		32160	96364	97620	-1	ISL3 family transposase
KCL43_00610		32160	98010	99206	-1	MFS transporter
KCL43_00615		32160	99794	100837	1	LPXTG cell wall anchor domain-containing protein
**GI-3**						
KCL43_02215		41007	450173	451570	-1	ISNCY family transposase
KCL43_02225		41007	451879	452958	-1	site-specific integrase
KCL43_02230		41007	453079	453567	-1	hypothetical protein
KCL43_02235		41007	453594	454676	-1	ImmA/IrrE family metallo-endopeptidase
KCL43_02240		41007	454677	455021	-1	helix-turn-helix transcriptional regulator
KCL43_02245		41007	455308	455517	1	XRE family transcriptional regulator
KCL43_02250		41007	455519	455704	1	helix-turn-helix transcriptional regulator
KCL43_02255		41007	455706	456482	1	phage antirepressor

KCL43_02260	41007	456541	456828	1	DNA-binding protein
KCL43_02265	41007	456825	456995	1	hypothetical protein
KCL43_02270	41007	457194	457403	1	hypothetical protein
KCL43_02275	41007	457484	457798	1	hypothetical protein
KCL43_02280	41007	457798	458955	1	DUF2800 domain-containing protein
KCL43_02285	41007	458968	459525	1	DUF2815 family protein
KCL43_02290	41007	459570	461492	1	DNA polymerase
KCL43_02295	41007	461496	463892	1	DNA primase
KCL43_02300	41007	464279	464554	1	VRR-NUC domain-containing protein
KCL43_02305	41007	464551	465870	1	DEAD/DEAH box helicase family protein
KCL43_02310	41007	465871	466038	1	hypothetical protein
KCL43_02315	41007	466035	466301	1	hypothetical protein
KCL43_02320	41007	466313	466933	1	site-specific DNA-methyltransferase
KCL43_02325	41007	467067	467480	1	transcriptional regulator
KCL43_02330	41007	467577	468029	1	terminase small subunit
KCL43_02335	41007	468019	469296	1	PBSX family phage terminase large subunit
KCL43_02340	41007	469312	470844	1	phage portal protein
KCL43_02345	41007	470804	472252	1	minor capsid protein
KCL43_02350	41007	472280	472468	1	hypothetical protein
KCL43_02355	41007	472471	472737	1	hypothetical protein
KCL43_02360	41007	472893	473462	1	DUF4355 domain-containing protein
KCL43_02365	41007	473475	474362	1	phage capsid protein
KCL43_02370	41007	474374	474730	1	phage head-tail connector protein
KCL43_02375	41007	474741	475019	1	hypothetical protein
KCL43_02380	41007	475016	475360	1	HK97 gp10 family phage protein
KCL43_02385	41007	475364	475723	1	hypothetical protein
KCL43_02390	41007	475735	476334	1	phage major tail protein, TP901-1 family

KCL43_02395	41007	476388	476843	1	hypothetical protein
KCL43_02400	41007	476918	477151	1	hypothetical protein
KCL43_02405	41007	477166	481323	1	tape measure protein
KCL43_02410	41007	481335	482177	1	phage tail family protein
KCL43_02415	41007	482187	484169	1	phage tail protein
KCL43_02420	41007	484169	484843	1	collagen-like protein
KCL43_02425	41007	484845	485459	1	hypothetical protein
KCL43_02430	41007	485473	487356	1	gp58-like family protein
KCL43_02435	41007	487365	487796	1	DUF1617 family protein
KCL43_02440	41007	487799	488413	1	DUF1366 domain-containing protein
KCL43_02445	41007	488426	488716	1	hypothetical protein
KCL43_02450	41007	488719	488898	1	hypothetical protein
KCL43_02455	41007	489019	490236	1	glucosaminidase domain-containing protein
KCL43_02460	41007	490577	490783	1	hypothetical protein
KCL43_02465	41007	490767	491180	1	DUF2335 domain-containing protein
**GI-4**					
KCL43_02705	9306	541569	542240	1	CPBP family intramembrane metalloprotease
KCL43_02710	9306	542237	542923	1	SagF family protein
KCL43_02715	9306	542946	543869	1	ABC transporter ATP-binding protein
KCL43_02720	9306	543878	545005	1	SagG family ABC transporter permease subunit
KCL43_02725	9306	545002	546120	1	ABC transporter permease
KCL43_02730	9306	546253	547659	-1	thioester-forming surface-anchored protein
KCL43_02735	9306	547981	549551	1	IS3 family transposase
KCL43_02740	9306	549660	551543	-1	thioester-forming surface-anchored protein
**GI-5**					
KCL43_02920	5454	597156	598931	1	hypothetical protein
KCL43_02925	5454	598928	599356	1	hypothetical protein

KCL43_02930		5454	599349	599753	1	hypothetical protein
KCL43_02935		5454	599802	600131	1	hypothetical protein
KCL43_02940		5454	600138	600836	1	TNT domain-containing protein
KCL43_02945		5454	600845	601249	1	hypothetical protein
KCL43_02950		5454	601439	601861	1	hypothetical protein
KCL43_02955		5454	601931	602539	1	hypothetical protein
KCL43_02960		5454	602899	603275	1	hypothetical protein
KCL43_02965		5454	603390	603626	1	hypothetical protein
KCL43_02970		5454	603727	604041	1	TNT domain-containing protein
KCL43_02975		5454	604059	604382	1	hypothetical protein
**GI-6**						
KCL43_03685		15589	762904	763185	1	hypothetical protein
KCL43_03690		15589	763263	764300	1	conjugal transfer protein
KCL43_03695		15589	764300	764785	1	PcfB family protein
KCL43_03700		15589	764782	766107	1	type IV secretory system conjugative DNA transfer family protein
KCL43_03705	ltrA	15589	766859	768769	1	group II intron reverse transcriptase/maturase
KCL43_03710		15589	768792	769382	1	TraM recognition domain-containing protein
KCL43_03715		15589	769463	770011	1	hypothetical protein
KCL43_03720		15589	770058	770276	1	hypothetical protein
KCL43_03725		15589	770336	770977	1	KilA-N domain-containing protein
KCL43_03730		15589	771116	771490	1	hypothetical protein
KCL43_03735		15589	771667	772872	1	ATP-binding protein
KCL43_03740		15589	773110	773265	1	single-stranded DNA-binding protein
KCL43_03745		15589	773709	774353	-1	histidine phosphatase family protein
KCL43_03750		15589	774372	774737	-1	YccF domain-containing protein
KCL43_03755		15589	774734	775213	-1	aminoacyl-tRNA deacylase
KCL43_03760	thiT	15589	775374	775937	-1	energy-coupled thiamine transporter ThiT

KCL43_03765	15589	776408	777274	-1	glycoside hydrolase family 25 protein
KCL43_03770	15589	777542	777994	1	hypothetical protein
KCL43_03775	15589	778206	778493	-1	hypothetical protein
**GI-7**					
KCL43_04770	4339	1004959	1005351	1	hypothetical protein
KCL43_04775	4339	1005520	1005846	1	hypothetical protein
KCL43_04780	4339	1006129	1006527	1	DUF1433 domain-containing protein
KCL43_04785	4339	1006529	1006774	1	hypothetical protein
KCL43_04790	4339	1006790	1007197	1	hypothetical protein
KCL43_04795	4339	1007207	1007497	1	hypothetical protein
KCL43_04800	4339	1007475	1007804	1	hypothetical protein
KCL43_04805	4339	1007806	1008057	1	hypothetical protein
KCL43_04810	4339	1008229	1008612	1	hypothetical protein
KCL43_04815	4339	1008609	1008869	1	hypothetical protein
KCL43_04820	4339	1008885	1009298	1	hypothetical protein
**GI-8**					
KCL43_05605	5946	1178693	1180051	-1	anion permease
KCL43_05610	5946	1180459	1180689	-1	hypothetical protein
KCL43_05615	5946	1180782	1181195	-1	hypothetical protein
KCL43_05620	5946	1181230	1181427	-1	helix-turn-helix transcriptional regulator
KCL43_05625	5946	1181867	1182070	-1	helix-turn-helix transcriptional regulator
KCL43_05630	5946	1182073	1182489	-1	hypothetical protein
KCL43_05635	5946	1182871	1183479	-1	DJ-1/PfpI family protein
KCL43_05640	5946	1183532	1184098	-1	GrpB family protein
KCL43_05645	5946	1184091	1184639	-1	DUF4368 domain-containing protein
**GI-9**					
KCL43_05820	9096	1223808	1225037	-1	transporter

KCL43_05825		9096	1225109	1225294	-1	hypothetical protein
KCL43_05830		9096	1225613	1225861	-1	Rgg/GadR/MutR family transcriptional regulator
KCL43_05835		9096	1225984	1227243	1	ISL3-like element ISSeq1 family transposase
KCL43_05840		9096	1227273	1227902	-1	Rgg/GadR/MutR family transcriptional regulator
KCL43_05850		9096	1228704	1229963	1	ISL3-like element ISSeq1 family transposase
KCL43_05855	rpsA	9096	1230000	1231202	-1	30S ribosomal protein S1
KCL43_05870		9096	1231581	1231811	-1	DUF2969 domain-containing protein
KCL43_05875		9096	1231879	1232904	-1	branched-chain amino acid aminotransferase
**GI-10**						
KCL43_06130		4547	1282244	1282456	-1	hypothetical protein
KCL43_06135		4547	1283297	1283590	-1	bacteriocin immunity protein
KCL43_06140		4547	1283620	1283841	-1	bacteriocin
KCL43_06145		4547	1284056	1284526	1	ATP-binding cassette domain-containing protein
KCL43_06150		4547	1284537	1285895	1	bacteriocin secretion accessory protein
KCL43_06155		4547	1285989	1286144	1	ComC/BlpC family leader-containing pheromone/bacteriocin
KCL43_06160	tnpA	4547	1286326	1286791	-1	IS200/IS605 family transposase
**GI-11**						
KCL43_06745		4314	1417395	1417574	1	helix-turn-helix transcriptional regulator
KCL43_06750		4314	1418207	1418530	-1	hypothetical protein
KCL43_06755		4314	1418531	1418923	-1	glycohydrolase toxin TNT-related protein
KCL43_06760		4314	1419223	1419969	-1	DUF1911 domain-containing protein
KCL43_06765		4314	1420575	1421204	-1	hypothetical protein
KCL43_06770		4314	1421287	1421709	-1	hypothetical protein
KCL43_06775		4314	1421691	1423724	-1	hypothetical protein
**GI-12**						
KCL43_07225		56229	1527778	1528980	1	ROK family transcriptional regulator
KCL43_07230		56229	1529143	1529472	-1	NUDIX domain-containing protein

KCL43_07235	56229	1529580	1531019	-1	recombinase family protein
KCL43_07240	56229	1531012	1532628	-1	recombinase family protein
KCL43_07245	56229	1532628	1534202	-1	recombinase family protein
KCL43_07250	56229	1534301	1534441	-1	hypothetical protein
KCL43_07255	56229	1534713	1534910	-1	helix-turn-helix transcriptional regulator
KCL43_07260	56229	1535110	1535273	1	transposase
KCL43_07265	56229	1535316	1537463	-1	ATP-binding cassette domain-containing protein
KCL43_07270	56229	1537545	1537880	-1	hypothetical protein
KCL43_07275	56229	1538277	1538810	-1	hypothetical protein
KCL43_07280	56229	1539087	1539830	-1	hypothetical protein
KCL43_07285	56229	1539955	1542099	-1	LPXTG cell wall anchor domain-containing protein
KCL43_07290	56229	1542503	1543982	-1	relaxase/mobilization nuclease domain-containing protein
KCL43_07295	56229	1544097	1545899	-1	group II intron reverse transcriptase/maturase
KCL43_07300	56229	1546472	1546864	-1	relaxase/mobilization nuclease domain-containing protein
KCL43_07305	56229	1546851	1547216	-1	MobC family plasmid mobilization relaxosome protein
KCL43_07310	56229	1547226	1547585	-1	hypothetical protein
KCL43_07315	56229	1547903	1550410	-1	AAA family ATPase
KCL43_07320	56229	1550452	1551213	-1	zeta toxin family protein
KCL43_07325	56229	1551213	1551689	-1	helix-turn-helix transcriptional regulator
KCL43_07330	56229	1551759	1552046	-1	hypothetical protein
KCL43_07335	56229	1552091	1552485	-1	chemotaxis protein
KCL43_07340	56229	1552489	1552713	-1	hypothetical protein
KCL43_07345	56229	1552767	1553853	-1	toprim domain-containing protein
KCL43_07350	56229	1553893	1554531	-1	peptidylprolyl isomerase
KCL43_07355	56229	1554713	1556227	-1	ABC transporter ATP-binding protein
KCL43_07360	56229	1556211	1557230	-1	radical SAM protein
KCL43_07365	56229	1557519	1558568	-1	radical SAM protein

KCL43_07370		56229	1558558	1558830	-1	PqqD family protein
KCL43_07375		56229	1558927	1559790	-1	helix-turn-helix domain-containing protein
KCL43_07380		56229	1560347	1560646	-1	hypothetical protein
KCL43_07385		56229	1560717	1561526	-1	helicase SNF2
KCL43_07390	ltrA	56229	1561660	1563318	-1	group II intron reverse transcriptase/maturase
KCL43_07395		56229	1563905	1564153	-1	PrgI family protein
KCL43_07400		56229	1564344	1565198	-1	type IV secretion system protein
KCL43_07405		56229	1565215	1565457	-1	hypothetical protein
KCL43_07410		56229	1565475	1567294	-1	type IV secretory system conjugative DNA transfer family protein
KCL43_07415		56229	1567441	1567701	-1	acetyl-CoA C-acyltransferase
KCL43_07420		56229	1567893	1568795	1	LysR family transcriptional regulator
KCL43_07425		56229	1569694	1570836	-1	hypothetical protein
KCL43_07430		56229	1570833	1571696	-1	ABC transporter ATP-binding protein
KCL43_07435		56229	1571707	1572033	-1	LytTR family transcriptional regulator DNA-binding domain-containing protein
KCL43_07440		56229	1572639	1572803	-1	hypothetical protein
KCL43_07445		56229	1573075	1573164	-1	DNA mismatch repair protein MutT
KCL43_07450		56229	1573285	1574022	-1	ABC transporter ATP-binding protein
KCL43_07455		56229	1574133	1576073	-1	prolyl oligopeptidase family serine peptidase
KCL43_07460		56229	1576098	1578728	-1	protein kinase/lanthionine synthetase C family protein
KCL43_07465		56229	1578777	1580387	-1	ABC transporter ATP-binding protein
KCL43_07470		56229	1580479	1581831	-1	FtsX-like permease family protein
KCL43_07475		56229	1581942	1582076	-1	SapB/AmfS family lantipeptide
KCL43_07480	era	56229	1583111	1584007	-1	GTPase Era
**GI-13**						
KCL43_07980		39852	1680841	1681506	-1	ComF family protein
KCL43_07985		39852	1681649	1681834	-1	Paratox
KCL43_07990		39852	1682132	1682485	1	hypothetical protein

KCL43_07995		39852	1682505	1682888	1	hypothetical protein
KCL43_08000		39852	1682872	1684131	1	hypothetical protein
KCL43_08005		39852	1684115	1685410	1	DUF3440 domain-containing protein
KCL43_08010		39852	1685394	1685906	1	ParB-like nuclease domain-containing protein
KCL43_08015		39852	1685950	1686153	1	hypothetical protein
KCL43_08020		39852	1686617	1686970	1	hypothetical protein
KCL43_08025		39852	1686970	1687767	1	recombinase family protein
KCL43_08030	acrIIA3	39852	1687928	1688281	1	anti-CRISPR protein AcrIIA3
KCL43_08035		39852	1688298	1688954	1	hypothetical protein
KCL43_08040		39852	1689032	1690246	-1	glucosaminidase domain-containing protein
KCL43_08045		39852	1690357	1690542	-1	hypothetical protein
KCL43_08050		39852	1690546	1690836	-1	hypothetical protein
KCL43_08055		39852	1690849	1691460	-1	hypothetical protein
KCL43_08060		39852	1691463	1691894	-1	DUF1617 family protein
KCL43_08065		39852	1691903	1693804	-1	gp58-like family protein
KCL43_08070		39852	1693814	1694428	-1	hypothetical protein
KCL43_08075		39852	1694430	1695104	-1	collagen-like protein
KCL43_08080		39852	1695104	1697161	-1	phage tail protein
KCL43_08085		39852	1697158	1697928	-1	phage tail family protein
KCL43_08090		39852	1697941	1702041	-1	phage tail tape measure protein
KCL43_08095		39852	1702267	1702569	-1	hypothetical protein
KCL43_08100		39852	1702662	1703237	-1	phage tail protein
KCL43_08105		39852	1703250	1703630	-1	hypothetical protein
KCL43_08110		39852	1703623	1704021	-1	HK97 gp10 family phage protein
KCL43_08115		39852	1704023	1704385	-1	phage head-tail adapter protein
KCL43_08120		39852	1704378	1704686	-1	hypothetical protein
KCL43_08125		39852	1704686	1704859	-1	hypothetical protein

KCL43_08130	39852	1704870	1706003	-1	phage major capsid protein
KCL43_08135	39852	1706020	1706826	-1	Clp protease ClpP
KCL43_08140	39852	1706807	1707994	-1	phage portal protein
KCL43_08145	39852	1708147	1708371	-1	hypothetical protein
KCL43_08150	39852	1708362	1710092	-1	terminase large subunit
KCL43_08155	39852	1710105	1710422	-1	P27 family phage terminase small subunit
KCL43_08160	39852	1710565	1710870	-1	HNH endonuclease
KCL43_08165	39852	1710863	1711249	-1	hypothetical protein
KCL43_08170	39852	1711521	1712096	-1	site-specific integrase
KCL43_08175	39852	1712199	1712600	-1	transcriptional regulator
KCL43_08180	39852	1712603	1712818	-1	hypothetical protein
KCL43_08185	39852	1712835	1713503	-1	site-specific DNA-methyltransferase
KCL43_08190	39852	1713524	1714111	-1	class I SAM-dependent methyltransferase
KCL43_08195	39852	1714115	1714342	-1	hypothetical protein
KCL43_08200	39852	1714335	1714535	-1	hypothetical protein
KCL43_08205	39852	1714532	1714831	-1	hypothetical protein
KCL43_08210	39852	1714873	1715262	-1	hypothetical protein
KCL43_08215	39852	1715326	1715538	-1	hypothetical protein
KCL43_08220	39852	1715547	1715687	-1	hypothetical protein
KCL43_08225	39852	1715684	1715917	-1	hypothetical protein
KCL43_08230	39852	1715898	1716281	-1	DnaD domain protein
KCL43_08235	39852	1716443	1716688	-1	transcriptional regulator
KCL43_08240	39852	1716892	1717095	-1	hypothetical protein
KCL43_08245	39852	1717354	1718070	-1	polymer-forming cytoskeletal protein
KCL43_08250	39852	1718215	1718430	-1	helix-turn-helix transcriptional regulator
KCL43_08255	39852	1718618	1719361	1	helix-turn-helix domain-containing protein
KCL43_08260	39852	1719421	1719891	1	hypothetical protein

KCL43_08265		39852	1719917	1720693	1	DUF1828 domain-containing protein
**GI-14**						
KCL43_09330		11583	1934565	1934780	1	transposase
KCL43_09335		11583	1935434	1935556	1	hypothetical protein
KCL43_09340		11583	1935560	1936777	-1	hypothetical protein
KCL43_09345		11583	1936979	1937851	1	hypothetical protein
KCL43_09350		11583	1937866	1938336	1	hypothetical protein
KCL43_09355		11583	1938425	1939744	1	DUF87 domain-containing protein
KCL43_09360		11583	1939834	1940148	1	hypothetical protein
KCL43_09365		11583	1940189	1940578	1	hypothetical protein
KCL43_09370		11583	1940752	1941546	1	Rep protein
KCL43_09375		11583	1941553	1941822	1	helix-turn-helix domain-containing protein
KCL43_09380		11583	1941865	1943085	1	tyrosine-type recombinase/integrase
KCL43_09385	rpsI	11583	1943193	1943585	-1	30S ribosomal protein S9
KCL43_09390	rplM	11583	1943605	1944051	-1	50S ribosomal protein L13
KCL43_09395		11583	1944578	1945438	-1	DegV family protein
KCL43_09400		11583	1945630	1946148	-1	NYN domain-containing protein
**GI-15**						
KCL43_09630		6817	1998820	1998924	1	putative holin-like toxin
KCL43_09635		6817	1999533	2000930	-1	ISNCY family transposase
KCL43_09640		6817	2001127	2002473	1	LPXTG cell wall anchor domain-containing protein
KCL43_09645		6817	2002766	2003039	1	YSIRK-targeted surface antigen transcriptional regulator
KCL43_09650		6817	2003281	2004264	-1	hypothetical protein
KCL43_09655		6817	2004531	2005637	-1	LPXTG cell wall anchor domain-containing protein

Table S5. Presence or absence of 14 selected putative virulence genes in *S. zooepidemicus* strains

An identity and coverage cutoff of 75% were used to determine virulence genes' presence or absence.

Isolates	*szP*	*mlpZ*	ATCC35246-type *szM**	*bifA*	*fszF*	*sdzD*	*spaZ*	*speK*	*speL*	*szeF*	*szeL*	*szeM*	*szeN*	*szeP*
2329	+	-	+	-	-	+	+	-	-	+	-	-	-	-
ATCC35246	+	-	+	+	+	+	+	-	-	-	-	-	-	-
AZ-45470	+	+	-	-	-	-	+	-	-	-	-	-	-	-
BHS5	+	-	-	-	-	-	+	-	-	+	-	-	+	+
CY	+	-	+	+	+	+	+	-	-	-	-	-	-	-
H70	+	-	+	-	-	+	+	-	-	-	-	-	-	-
IA50014PP30511	+	+	-	-	+	+	+	-	-	-	-	-	-	-
IA-61192A	+	-	+	+	+	+	+	-	-	-	-	-	-	-
IA-61192B	+	-	+	+	+	+	+	-	-	-	-	-	-	-
IN-6992	+	+	-	-	+	+	+	-	-	-	-	-	-	-
ISU16140	+	+	+	-	-	+	+	-	-	-	-	-	-	-
ISU36185	+	-	+	-	-	+	+	-	-	-	-	-	+	+
ISU37775	+	+	-	-	+	+	+	-	-	-	-	-	-	-
ISU38408	+	-	-	-	+	+	+	-	-	+	-	-	-	-
ISU54026	+	-	+	-	-	+	+	-	-	-	-	-	-	-
ISU54485	+	-	+	-	-	+	+	-	-	-	-	-	-	-
ISU6659	+	+	+	-	-	+	+	-	-	-	-	-	-	-
ISU75596	+	-	+	-	-	+	+	-	-	-	-	-	+	-
ISU88977	+	+	+	-	-	+	+	-	-	-	-	-	-	-
ISU9714	+	-	+	-	-	+	+	-	-	-	-	-	-	-
MGCS10565	+	+	-	-	+	+	+	-	-	-	-	-	-	-

NCTC11606	+	+	-	-	+	+	+	-	-	-	-	-	-	-
NCTC11824	+	+	-	-	-	-	+	-	-	-	-	-	-	-
NCTC12090	+	-	+	-	-	+	+	-	-	-	-	-	+	+
NCTC4676	+	-	-	-	-	+	+	-	-	-	-	-	-	-
NCTC6176	+	+	-	-	-	+	+	-	-	-	-	-	-	-
NCTC6180	+	-	-	-	-	-	+	-	-	+	-	-	-	-
NCTC7022	+	-	-	-	-	-	+	-	-	+	-	-	-	-
NCTC7023	+	-	-	-	-	-	+	-	-	+	-	-	-	-
NVSLBI19	+	-	-	-	-	-	+	-	-	-	-	-	+	-
OH-71905	+	-	+	-	+	+	+	-	-	-	-	-	-	-
S31	+	-	-	-	-	-	-	-	-	+	-	-	-	-
S32	+	+	-	-	+	+	+	-	-	-	-	-	-	-
S33	+	+	-	-	+	+	+	-	-	-	-	-	-	-
S34	+	+	-	-	+	+	+	-	-	-	-	-	-	-
Sz105	+	+	+	-	-	+	+	-	-	-	-	-	-	-
Sz12is	+	-	+	-	+	-	+	-	-	-	-	-	-	-
Sz16	+	+	-	-	+	-	+	+	+	-	+	+	-	-
Sz35	+	+	-	+	+	-	-	-	-	-	-	-	-	-
Sz4is	+	-	+	-	+	-	+	-	-	-	-	-	-	-
Sz5	+	+	-	-	-	-	+	+	+	-	+	+	-	-
Sz57	+	-	-	-	-	+	+	-	-	+	-	-	-	-
SzAM35	+	+	-	-	+	-	+	-	-	-	-	-	-	-
SzAM60	+	-	-	-	-	-	+	-	-	-	-	-	-	-
Szoo-S19	+	-	+	+	+	+	+	-	-	-	-	-	-	-

Szoo-S2	+	-	+	+	+	+	+	-	-	-	-	-	-	-
Szoo-S22	+	-	+	+	+	+	+	-	-	-	-	-	-	-
SzS31A1	+	-	+	-	+	-	+	-	-	-	-	-	-	-
TN-714097	+	-	+	-	+	+	+	-	-	-	-	-	-	-
TN-LIVER4	+	-	+	+	+	+	+	-	-	-	-	-	-	-
TN-LUNG1	+	-	+	+	+	+	+	-	-	-	-	-	-	-
TN-LUNG2	+	-	+	+	+	+	+	-	-	-	-	-	-	-
TN-LUNG3	+	-	+	+	+	+	+	-	-	-	-	-	-	-
TN-TB1	+	-	+	+	+	+	+	-	-	-	-	-	-	-
TN-TC1	+	-	+	+	+	+	+	-	-	-	-	-	-	-

* An identity and coverage cutoff of 75% were used to determine the presence of ATCC35246-type *szM* gene (GenBank accession #:AEJ26159.1).

Table S6. Information of 14 selected putative virulence genes in *S. zooepidemicus*

bifA	AEJ25307.1	phosphoribosylaminoimidazole-succinocarboxamide synthetase [Streptococcus equi subsp. zooepidemicus ATCC 35246]
mlpZ	ACG61714.1	M-like protein [Streptococcus equi subsp. zooepidemicus MGCS10565]
szeF	AGS46828.1	exotoxin F, partial [Streptococcus equi subsp. zooepidemicus]
szeN	AGS46830.1	exotoxin N [Streptococcus equi subsp. zooepidemicus]
szeP	AGS46829.1	exotoxin P, partial [Streptococcus equi subsp. zooepidemicus]
szM	AEJ26159.1	antiphagocytic cell surface-anchored fibrinogen-and IgG Fc-binding protein SzM [Streptococcus equi subsp. zooepidemicus ATCC 35246]
szP	AAQ08507.1	Szp protein [Streptococcus equi subsp. zooepidemicus]
	AGE09604.1	Szp protein, partial [Streptococcus equi subsp. zooepidemicus]
	AAQ08496.1	Szp protein [Streptococcus equi subsp. zooepidemicus]
	AAQ08500.1	Szp protein [Streptococcus equi subsp. zooepidemicus]
	AGE09605.1	Szp protein, partial [Streptococcus equi subsp. zooepidemicus]
	AGE09606.1	Szp protein, partial [Streptococcus equi subsp. zooepidemicus]
	AAQ08506.1	Szp protein [Streptococcus equi subsp. zooepidemicus]
	AAC43319.1	M protein precursor; MSzW60 [Streptococcus equi subsp. zooepidemicus]
szeL	CAH65000.1	exotoxin L [Streptococcus equi subsp. zooepidemicus]
szeM	CAH68555.1	exotoxin M [Streptococcus equi subsp. zooepidemicus]
fszF	AEJ26208.1	fimbrial subunit protein [Streptococcus equi subsp. zooepidemicus ATCC 35246]
	ACG63156.1	fimbrial subunit protein FszF [Streptococcus equi subsp. zooepidemicus MGCS10565]
sdzD	ACG62033.1	streptodornase D type, SdzD [Streptococcus equi subsp. zooepidemicus MGCS10565]
spaZ	ACG61542.1	protective antigen-like protein, fibrinogen- and Ig-binding protein precursor [Streptococcus equi subsp. zooepidemicus MGCS10565]
speK	WP_11054728	streptococcal exotoxin SpeK - phage associated [Spes (VF0248) - Exotoxin (VFC0235)] [Streptococcus pyogenes MGAS10270]
	WP_41174277	streptococcal exotoxin SpeK - phage associated [Spes (VF0248) - Exotoxin (VFC0235)] [Streptococcus pyogenes SSI-1]
speL	WP_11017837	streptococcal exotoxin L precursor [Spes (VF0248) - Exotoxin (VFC0235)] [Streptococcus pyogenes MGAS8232]
